# Review: DNA Barcoding and Chromatography Fingerprints for the Authentication of Botanicals in Herbal Medicinal Products

**DOI:** 10.1155/2017/1352948

**Published:** 2017-04-27

**Authors:** Bashir Mohammed Abubakar, Faezah Mohd Salleh, Mohd Shahir Shamsir Omar, Alina Wagiran

**Affiliations:** ^1^Department of Biotechnology & Medical Engineering, Faculty of Biosciences and Medical Engineering, UTM, 81310 Skudai, Johor, Malaysia; ^2^Department of Biological Sciences, Bauchi State University Gadau, PMB 065, Bauchi, Nigeria; ^3^Department of Biosciences & Health Sciences, Faculty of Biosciences and Medical Engineering, UTM, 81310 Skudai, Johor, Malaysia

## Abstract

In the last two decades, there has been a tremendous increase in the global use of herbal medicinal products (HMPs) due to their claimed health benefits. This has led to increase in their demand and consequently, also, resulted in massive adulteration. This is due to the fact that most of the traditional methods cannot identify closely related species in a process product form. Therefore the urgent need for simple and rapid identification methods resulted in the discovery of a novel technique. DNA barcoding is a process that uses short DNA sequence from the standard genome for species identification. This technique is reliable and is not affected by external factors such as climates, age, or plant part. The difficulties in isolation of DNA of high quality in addition to other factors are among the challenges encountered using the DNA barcoding in the authentication of HMP. These limitations indicated that using DNA barcoding alone may ineffectively authenticate the HMP. Therefore, the combination of DNA barcoding with chromatographic fingerprint, a popular and generally accepted technique for the assessment and quality control of HMP, will offer an efficient solution to effectively evaluate the authenticity and quality consistency of HMP. Detailed and quality information about the main composition of the HMPs will help to ascertain their efficacy and safety as these are very important for quality control.

## 1. Introduction

In the last two decades, there was a tremendous increase in the global use of herbal medicinal products (HMPs) [1]. These general increases in the consumption of HMP are due to their claimed health benefits, easy availability, perceived effectiveness, and safety [2, 3]. HMP generally referred to any medicinal product that exclusively contained active ingredient herbal substance (part of the plant) or herbal extract [4]. In the context of this paper HMPs, or herbal medicines, encompass both simple preparation and manufactured products containing one or more herbal active ingredients.

It is estimated that 5.6 billion people, approximately, 80% of the world population, rely on HMPs for their primary health care [5–9]. More than 90% of Africans as well as 70% and 40% of Indian and Chinese population, respectively, continue to rely on HMP for general health care [10]. Despite the great advances achieved in the field of modern medicine, plants still play a significant role in health care. At least, 25% of drugs in modern pharmacopeia are directly or indirectly derived from plants [11, 12] (Palhares et al., 2015a) and more than 60% of antitumour drugs are generally derived from natural product [13]. The international trade of HMPs is becoming a lucrative business due to their high demand, their global market is estimated at US$83 billion (Palhares et al., 2015a), and it is expected to reach US$107 billion by 2017 [3].

The increase in usage of these HMPs is not only restricted to developing countries; however developed countries such as USA, Italy, France, and Germany have produced guidelines for the registration of herbal medicine [11]. The WHO reported that the annual sales of herbal medicine in Germany for the year 2002 have reached US$2.432 billion [14]. Nahin et al. [15] also reported that the market of HMPs in USA has expanded tremendously from US$2.9 billion to US$4.8 billion from 1995 to 2008.

So, also, the average import of these HMPs in USA in 2004–2008 is more than US$220 million and the highest exporters for that very period were China and India valued at around US$348 and US$93 million, respectively [16]. After a period of 3 years, the sales of herbal medicine in China increased tremendously as reported by National Bureau of Statistics [14]. They reported that the sale value in herbal industries has reached US$68 billion (RMB418 billion) with an annual growth rate of 37.9%. So, also, the export annual rate of the herbal medicine from China to US in the same years has reached up to 66.3% [17].

Despite the extensive usage of HMP for a long period of time, issues related to their assessment and quality control are always a major problem because active phytocompounds have inherent risk just like all the active chemical compounds [18]. The problems encountered are mostly attributed to the poor quality of raw material or finished products and may result in different types of adverse effects. Basically, the quality of the herbal products can be categorised into external and internal categories [19]. The external issue affecting the quality includes contamination, cultivation, processing, adulteration, and misidentification while internal issues are basically caused by the presence of bioactive chemicals in the herbal products.

Both the external and internal qualities of the raw material are very important due to the fact that both safety and efficacy of the herbal products depend on them. According to the WHO, adulteration of the herbal product is not only a fraud but a threat to the consumer's health as this might result in adverse effects and eventually death [20]. False identification of HMPs has resulted in several toxic effects to consumers. A dramatic example of such incident occurs in an 18-month-old baby who was diagnosed with venoocculusive disease after regularly consuming a herbal tea containing high amount of pyrrolizidine alkaloid [21]. Other cases include adulteration of herbal medicine tea with a neurotoxin compound from* Illicium anisatum* [22].* Aconitum carmichaelii* and* Aconitum kusnezoffii* have long been used to relief pain conditions such as rheumatism, arthritis, bruises, and fractures [23]. Most of the toxicity of these HMPs is primarily derived as a result of the presence of diterpene alkaloids particularly aconitine; others include mesaconitine and hypaconitine [24]. In traditional Chinese medicine (TCM) practices, aconitine has been effectively used to treat various ailments despite their toxic effects. Such effects were related to that causing ventricular tachycardia [25], bradycardia, and hypotension [26].

The proportion of adulterated HMPs sold in developing countries can account for up to 10% but may increase up to 50% when purchased through unregulated channels such as online transaction [27]. The popular demand in such market especially in developing countries may pose further a serious problem where drug regulation is weak. Reports have showed that more than 80% of the HMPs sold in Africa are counterfeit or adulterated [28]. All these incidences keep compounding and increasing frequently due to lack of proper identification methods for primary plant source [27].

In general, HMPs are classified into two products: monoherbal and polyherbal products [29]. Monoherbal products contained only one herb or herbal component, for example,* Eurycoma longifolia* capsule which consists of its extract only. The polyherbal normally can be found either in the form of capsule, tablets, or tea which consists of more than one (two or more) herb component which can work in synergy to deliver their therapeutic effects. Liuwei Dihuang pill (LDP), a well known TCM widely used for the treatment of kidney yin deficiency, contains 6 different Chinese medicinal herbs [30]; Hwangryunhaedok-tang (HHT), a traditional herbal medicine that is used for the treatment of inflammation, fever, hypertension, and gastritis in China and Japan, is composed of four herbal substances [31]. Other examples of polyherbal products are shown in Table 1.

Due to the widespread use of medicinal plants for the past decades tandem with the assurance of its quality, safety, and efficacy of medicinal plant, the WHO has published monographs [32–35] for selected medicinal plants for references. Each volume of these monographs consists of different species of plant with their synonyms and vernacular names, commonly used parts, geographical location, known active compounds, dosage forms, potential adverse effects, contraindication, recognised medicinal benefits, and how to correctly and effectively use them. Some countries have also established a series of monographs for their local usage such as Chinese pharmacopeia [36], United States pharmacopeia [37], Ayurvedic Pharmacopoeia of India [38], Japanese pharmacopeia [39], and other countries. The main purpose of developing all these monographs is to provide accurate scientific information on the safety, efficacy, and quality assurance of the widely used medicinal plants and to help other countries to develop their own.

Certain steps are of enormous importance in order to guarantee the quality of HMPs and these include (1) correct identification of the plant species and (2) correct identification and analyses of the purity of the pharmacologically active constituent (biomarkers with their required minimum concentration) [40]. In this aspect, the identification of the exact and correct raw material used for the production of HMPs is always challenging. Correct identification of the source of raw materials of medicinal herbal plants used is the prerequisite and is of utmost criticalness to ensure their quality, safety, and therapeutic efficacy [41, 42]. Generally, there are different types of identification methods, most are commonly based on botanical and morphological analyses by taxonomist expert; thus identification by common people may lead to incorrect specie. This is also true in the sense that most of the HMP consists of dried or unidentifiable part and this makes the species identification process difficult even to highly trained and experienced professional taxonomist [27].

The identification of herbal medicinal plant for quality control and standardization of raw materials should comply with the requirement specified in pharmacopeia; these include organoleptic evaluation (identification of senses: touch, smell, sight, and taste), morphological characteristics such as microscopic and macroscopic (shape, colour, and texture), and chemical profiling such as HPLC, TLC, and GC [43]. First of all, if the identity of the plant part is verified, the process then proceeds with chemical analysis using the chromatographic methods such as HPLC so as to identify or to determine the profile of the active compounds (active/biomarkers) of the product and to quantify them (Palhares et al., 2015a) [43].

However, these methods have their own limitations as the former methods primarily depend on human expertise [27]; the latter may be affected by several factors such as age, growth, and storage condition [42]. These methods cannot identify closely related species which shared similar or identical morphological characteristics and chemical profiles. This indicates that chemical profiling techniques can only detect an indirect fraud as it lacks the temerity to reveal the identity of the adulterated species. However, this shows that neither of these methods can distinguish between closely related species; therefore, there is an urgent need to introduce an accurate, simple, and rapid identification method for proper inspection of HMPs.

In addition to other identification methods, DNA barcoding can serve as a powerful tool to overcome some of the problems encountered. DNA barcoding is a process which involves the use of short DNA sequence from the standard part of the genome for species identification. This unique technique was first coined by Canadian zoologist Herbert in 2003; he used the 5′ end of cytochrome C oxidase subunit of mitochondria to identify closely related allies of lepidopterans [44]. Contrary to chemical profiling, DNA barcoding can provide a reliable and consistent result because it is not influenced by sample age, environmental factors, physiological condition, growth condition, harvesting period, cultivation area, plant part, and storage condition [42, 45].

The species-level discrimination of DNA barcoding has made this technique widely accepted as a regulatory tool for evaluating the authenticity of different types of HMPs [20, 46–48]. The authentication of HMPs is made possible using DNA barcoding because DNA is more reliable, is stable in contrast to other macromolecules (e.g., RNA) as it is not affected by internal or external factors, and is found in all tissues [45]. The stability of DNA makes it possible to identify species in a dried or process product form against the morphological features of the plant on which the current Linnaean taxonomic system is based on [20]. Moreover, despite the fact that DNA barcoding serves as an efficient method for identifying and authenticating HMPs, it is also characterised by some limitations [49, 50].

For these reasons, it can be observed that none of the methods are 100% fit for authentication and evaluation of the quality of the HMP. This review tends to highlight the need to combine both DNA barcoding and chromatographic fingerprint in order to effectively validate the authenticity and evaluate the quality of the HMPs. Detailed knowledge about the main composition of the HMPs will help to ascertain their efficacy and safety for quality purpose control.

## 2. High Performance Liquid Chromatography (HPLC)

Chromatography is a technique used for the separation of a mixture into their constituent component. This technique was first coined by a Russian botanist, Mikail Tswett, in 1903 when he separated plant pigments such as chlorophyll and xanthophylls by passing their solution through a glass column packed with finely divided calcium carbonate [51]. As the plant pigments are coloured, the separation was named using two Greek words “Chrome” and “graphein” meaning “colour” and “to write,” respectively. After the invention, the value of his work was not appreciated for a few years, but, after a few decades, Tswett discovery was reconsidered leading to an explosive emergence of various modalities of chromatography. The complex mixtures are separated due to the difference in their time taken for each component to travel through a system that contains an immiscible bed of material or stationary phase and mobile phase [52]. The mobile phase transports analyte while the stationary phase is immobile. There are different types of chromatography and these are classified based on the physical state of the mobile phase, such as gas chromatography (GC) for gas mobile phase while liquid mobile phase is known as liquid chromatography (LC) [53].

Due to the versatile application of these techniques, chromatography fingerprints are now widely used in the analysis of complex compounds such as HMPs for quality control including authentication processes and efficacy and safety evaluation [54]. Chromatography fingerprint in herbal medicine is defined as the study of the chromatographic pattern of pharmacological active compounds present in the herbal medicine under study [55] and has been accepted by WHO since 1991 [56, 57].

However, among the different types of chromatography fingerprint that exist, the high performance liquid chromatography (HPLC) is undoubtedly one of the most popular and widely used chromatography fingerprints for the analysis of herbal medicines [58–62]. Since 1991, the WHO has accepted this technology as a strategy for identification and quantification of herbal medicine [56, 57]. Qualitatively, HPLC provides information about the presence of compounds and quantitatively the actual amount of that compound is present in the herbal extract or finished HMP. The qualitative and quantitative identification of the unknown compounds is done by comparing their HPLC retention times with unknown and UV spectra [63].

High reproducibility, sensitivity, selectivity, and the ability to analyse a number of constituents in HMP are among the great advantages of using HPLC techniques [18, 64]. However, for effective HPLC operation and to achieve the desired objective, certain important factors have to be taken into consideration starting from sample preparation to the final analysis.

### 2.1. Sample Preparation

Sample preparation is a crucial aspect in any analytical process as it occupies around two-thirds of the time of the whole process and accounts for almost one-third of the errors generated [158, 159]. It is therefore a very important part of chemical/biological analysis as it assists in removing potential interference and the analyte becomes detectable [160]. Sample preparation is also important in HPLC analysis as it helps to remove analytes from its original matrix into one that is homogenous and reproducible for proper analysis [161]. Complete dissolution of the sample in the eluent subsequently followed by filtration using a microfilter (usually with 0.45 *μ*mM) are the fundamental steps before HPLC analysis. This is very important because it will help to produce a solution that will be suitable for injection into the column during HPLC analysis. The overall outcome of any HPLC analysis is solely dependent on the quality of the sample preparation used. There are different methods of sample preparation that are necessary to modify the sample amenable for HPLC analysis so as to improve the result of the analysis. This can involve a number of operations that may be necessary to modify the samples, and this may also have various purposes, for example, sample fractionation, cleanup, and concentration of the analytes.

Before embarking on the normal operation of sample preparation of HMP for HPLC analysis, there exists a routine sample manipulation which is weighing the sample. It is very important to know the appropriate amount of sample that will be taken into the analysis as this may affect the analysis result as much as the final output of the chromatographic measurement. Other basic operation techniques to be used for the HPLC analysis depend largely on the nature of the sample matrix to be analysed either as solid, semisolid, liquid, or gas [159]. For example, simpler sample preparation is employed if the sample matrix is soluble in the mobile phase. However, in a situation where the sample matrix is not suitable in the mobile phase as in the case of powder or herbs, a suitable extraction procedure should be applied. It is very important that the chosen extraction method should contain low molecular constituent of herbal products, and this is mostly achieved by the use of methanol and ethanol. Some of the basic extraction protocols or operations in sample preparation of HMP for HPLC analysis include homogenisation of the sample matrix, dissolution, and filtration.

#### 2.1.1. Sample Homogenisation/Size Reduction

This is one of the most used steps in the preparation of HMPs for HPLC analysis due to their physical nature of the solid sample matrix. This is usually achieved by reducing the sample matrix size into a fine powder using pestle and mortar [162] or any other laboratory mills or blender. The ground material is then transferred into a volumetric flask for further extraction.

#### 2.1.2. Extraction Technique

Extractions are mostly performed at room temperature due to the fact that most analytes have aqueous solubility or a mixture of aqueous and organic media [163]. Extraction can be done in one step with single solvent or in multiple numbers of steps with numerous solvent depending on the nature of the analytes. Information about the nature of the analytes (at least class of the analytes such as organic, inorganic, ionic character, or functional group of the organic compound) to be measured is of vital importance. This is due to the fact that the solvent to be used for the extraction process must be able to solubilize the analytes and must be compatible with the HPLC mobile phase for the final analyses. Other qualities of solvent include high purity, compatibility with the detector, and low viscosity to keep the system back pressure low [164]. Furthermore, the dissolved oxygen present in the solvent may lead to system instability but can be removed by degassing step.

Dissolution of the analytes can enhance the extraction process and this can be achieved by agitation of the whole HMPs (e.g., capsule or grounded powder) by the use of ultrasound machine. Ultrasonic extraction process is the most used extraction processes for HMPs [30, 165–167] and this has more benefits than other techniques [168]. Fast extraction rates, better solvent penetration, greater yield extraction, maintaining low temperature, increased mass transfer, and less dependency on solvent are all the features that make sonication more advantageous than other types of technique [169–171].

Other methods used include mixing, shaking, inversion, and vortexing [71]. Sonication process facilitates the transfer of analytes from the sample matrix to the solvent and this is generally achieved in a short period of time [172]. Methanol is the most commonly used solvent in the sonication process of HMP extraction [70, 86, 173, 174]. Other sample preparation techniques include solvent immersion, solvent partitioning, refluxing, solid-phase microextraction, supercritical-fluid extraction, pressurized-liquid extraction, microwave-assisted extraction, solid-phase extraction, and surfactant-mediated extraction.

The extracted analytes of the HMP are further filtered using microfilter (normally 0.45 *μ*m) before being subjected to HPLC analysis as reported by several studies [175, 176] and this is a crucial step because it may remove any particulate which may block the column or interfere with the HPLC analysis.

### 2.2. Mode of Separation: Mobile and Stationary Phase

HPLC is one of the most popular methods for the analysis of any crude mixtures such as HMPs and this requires either reverse-phase or normal-phase chromatographic mode [164, 177]. The mobile phase is the liquid phase of HPLC which carries the analytes and continues flowing through the stationary phase, the phase where the analytes are separated by adsorption. The use of any mode (mobile phase) depends on the compatibility of the stationary phase with the nature of the extract or mixture to be analysed. So, also, the mode to be used also depends on the chemical nature of the crude extract.

It is an easy method which generally requires either reverse-phase or normal phase whose choice of usage depends on the compatibility of the stationary phase with the mixture or extract to be analysed. The method to be used also depends on the chemical nature of the crude extract. Among the two, the reverse-phase column is the most commonly used method for the separation of HMP when compared to the normal phase and is always more likely to produce a satisfactory final result [178].

#### 2.2.1. Normal-Phase HPLC (NP-HPLC)

The normal-phase HPLC (NP-HPLC) which was formally known as adsorption HPLC is not the most popular form of HPLC analysis for analysis of HMPs. This method is best used for analysis of polar analytes as it utilizes a polar stationary phase which usually consists of silica compared to a nonpolar mobile phase, for example,* n-hexane*. This is due to the fact that the polar compounds will strongly bind or be adsorbed to the polar silica in the column, making the nonpolar compound quickly pass through the stationary phase. Increasing the polarity of solvent in the mobile phase will decrease the elution (retention) time of the analytes. On the other hand, more hydrophobic solvent will decrease the retention time. The popularity of using NP-HPLC decreased in 1970 with the development of reverse phase (RP-HPLC) due to the lack of reproducibility and retention times [179].

The eluents that are mostly used in NP-HPLC mostly consist of a mixture of different types of hydrocarbons such as aliphatic (e.g., n-hexane and n-heptane), halogenated (e.g., chloroform), more polar oxygenated (e.g., ethyl acetate, diethyl acetate, and acetone), or hydroxylated solvents (e.g., isopropanol and methanol) [164].

#### 2.2.2. Reverse-Phase HPLC (RP-HPLC)

Reverse-phase HPLC (RP-HPLC) is the most commonly used HPLC mode that is used for the analysis of HMP [18, 61]. As the name implies, it is just the reverse mode of NP-HPLC. The selection of stationary phase is one of the most important factors for fingerprint development [180]. Commonly used RP-HPLC stationary phase for herbal fingerprint is the silica which is modified or treated with nonpolar RMe_2_SiCl; R is a straight chain alkyl group where the long chain (e.g., C_18_H_37_) is more hydrolytically stable than short chain group (e.g., C_8_H_17_) [177, 181]. Typically, 2–5 *μ*m particle size is used as this is very important for maintaining their efficiency; backpressure is drastically increased if small particle size is used [29, 180].

In contrast with NP-HPLC, RP-HPLC has a broad range of selectivity coupled with a high degree of reproducibility [177]. The retention time of RP-HPLC is longer for less polar molecules; as such, the polar molecules elute more readily. So, also, most of the eluents used in the RP-HPLC are composed of water mixed with miscible organic solvents which are mostly acetonitrile, methanol, or tetrahydrofuran [164]. PH also plays a vital role as it changes the hydrophobicity of the analytes. For this reason, buffer, acids, or bases are also added in order to control the PH. The effects of buffer and acids differ tremendously by application but generally improve chromatography result [179].

### 2.3. Isocratic Flow and Gradient Elution

The term isocratic means constant composition [179]. It is a type of separation analysis in which the mobile phase composition is constant throughout the HPLC analysis; as such any equilibration of the column is not required. Isocratic flow elution is always suitable for separation of mixture with few compounds. Isocratic flow with commonly used reverse-phase column is also used in the analysis of HMP [71, 173, 182, 183]. In terms of gradient elution, it is the reverse case of isocratic analysis as the mobile composition changes overtime. Gradient elution with reverse-phase is the most commonly used in the HPLC analysis of multiple constituent present in herbal preparation or medicinal plants [184–189].

### 2.4. Detectors

A detector which consists of a sensor and electronics is a device used with HPLC to detect or monitor the presence of sample components in a medium, while eluting the column. After the columns have performed their actual role of separation, the next task is to identify the presence of the separated component in the sample and this function is achieved using detectors. This situation makes the detector the second most important component of HPLC analysis as it will be meaningless to separate components in a sample without identifying them. The detection of chemicals using HPLC analysis is possible as a result of a specific physiochemical property of the sample which is different from that of the mobile phase. The detector provides an electric signal which is proportional to the quantity or concentration of the component within the detector at a given moment. The signals which are produced in the form of peaks are then further processed by passing them into an integrator, which will be used to quantify the amount of the components present. The use of peak areas in the chromatogram is the most common way of quantification [190].

The criteria for a specific choice for the selection of detectors depend on some factors which include sensitivity of the detector for a specific property, difference in properties from that of the mobile phase, and separation condition used for the analysis. The detection of molecules after eluting them from the chromatographic column can be achieved using varieties of principles and techniques, and this can be classified as those that can detect all sample components, particular group of compound, or only one compound [191]. The first group which can response to all samples components are called universal and a good example of such detector is the UV/Vis detector. The second are known as selective while the last are specific.

The various detection techniques used for the HPLC analysis of HMP include the Ultraviolet-Visible (UV-Vis) or photodiode-array, the evaporative light scattering (ELS), and the spectrometry (MS) as well as other types of other detection techniques. However, as detectors are very crucial in HPLC analysis, they are expected to possess some qualities which include sensitivity, linearity in a wide range of sample, reproducibility in the response, stability to changes in flow, and environmental parameters and capability of detection in a small volume of sample [190]. In general, the most frequently used detectors for the analysis of HMP are the UV-Vis [71, 80] and ELSD [75, 192–194].

UV/Vis is a universal detector used in HPLC system and was first described by Horvath and Lipsky in 1966 [195]. This type of detector is capable of absorbing electromagnetic radiation in UV/Visible wavelength range of 190–600 nm. It is extremely important as it gives room for analysis of natural products containing chromophores such as flavonoids, coumarin, or isoflavin. The UV/VIS detectors are easy to operate, particularly suitable for less skilled operators. The diode-array detector (DAD is also known as photodiode-array, PDA) is an advanced form of UV/Vis that is frequently used for the analysis of HMP coupled with an HPLC to form hyphenated technique HPLC-PDA also known as LC-PDA [72, 79, 196]. The PDA provides more information on sample composition than any other detectors due to the fact that it can collect data at one or more wavelength across a chromatogram in one or more analytes in a single run [64]. However, the inherent limitation of using PDA UV/VIS detector is that it can only absorb compounds that have absorbing chromophores; those that do not have them will, therefore, have little or no effects on the UV detector [61, 197]. PDA is one of the most commonly used detectors utilized for the screening of vitamin, drugs, natural products, and herbal medicines [198], probably due to its high sensitivity to many solutes, simplicity, and low cost [29].

The ELSD is also a universal type of detector used for the HMP analysis. As a universal detector, it has the potential to be used for any type of sample including those that do not have good light absorbance in the UV. The work of this type of detector begins with nebulization of the solution eluted from the column to form aerosol that is further converted to larger droplets so as to enhance detection [199]. The only requirement for using this type of detector technique is that the component of the sample should be less volatile than the component of the mobile phase [195]. ELSD has also played a vital role in the analysis of compounds with little or weak chromophores such as saponins [57], aglycone [200], terpenes [83], glycosidic form [193], and other forms of alkaloids [201] which may be difficult to detect using UV detector. However, nonlinearity of ELSD as reported by some researchers (Verhelst and Vandereecken [202], Carbognani [203]) is among the drawbacks or limitations of using this type of detector in the analysis of complex mixture of compounds.

Due to some limitations of HPLC-UV-Vis or HPLC-PDA, nowadays HPLC coupled with mass spectrometry (MS) has become a powerful tool for the qualitative and quantitative analysis of complex mixture such as HMP or natural product extract [204]. This is due to the fact that hyphenated MS with HPLC as a detector has high sensitivity and selectivity for the analysis of natural product HMP. The term “hyphenated” refers to coupling of an independent analytic instrument to provide detection. In HPLC-LC, also known as LC-MS, the samples separated from the column can be observed in the form of spectral data [196]. Atmospheric-pressure ionization (API) interfaces, which include electrospray ionization (ESI) and atmospheric-pressure chemical ionization (APCI), are used to overcome the problem of high mobile phase volume during the HPLC analysis and it is the most successful interface used in HPLC-MS configuration [63, 205]. The use of interfaces together with MS in the analysis of HMP has become a powerful tool due to its high sensitivity, low level of sample consumption, and rapid analysis time [206] Most of these ionization techniques used in this detection technique (HPLC-MS) are generally soft ionization techniques and this does not typically produce many fragments.

The use of LC-MS in the analysis of HMP has now attracted a lot of attention compared to any type of detection method due its high sensitivity and selectivity. As mentioned earlier, the introductions of the various types of interface more especially ESI and APCI with respect to the analysis of HMP have also made this type of detection technique extremely important. This is due to the fact that with the presence of these interfaces, various analysers such as quadrupole (Q) ion trap (IT) and time of flight (TOF) can also be used and all these conferred a better advantage in the qualitative and quantitative analysis of the HMP. However, upon the various advantages of using this type of hyphenated technique in the analysis of HMP, the quality of response strongly depends on the nature of the compound to be used, the type of solvent and buffer to be used in the mobile phase, the type of interface to be used, and so on. All these factors are problems associated with the use of this type of detection technique.

Other HPLC hyphenated techniques such as NMR spectroscopy are available for the analysis of HMP. The HPLC-NMR or LC-NMR which has been in existence for the past two decades helps in providing useful information about the structure elucidation of natural product [196]. However, upon the various advantages of using this type of detection technique in the analysis of natural product, LC-NMR has not become a widely accepted hyphenated technique for HPLC analysis and this could be attributed as a result of its low sensitivity. The summary of the different types of the detection technique used in the analysis of the HMP is shown in Table 2.

The qualitative and quantitative analysis of the HMP are very significant in the authentication of HMPs as they serve as the prerequisite for disclosing their overall benefits and risk [206]. The developments of the modern analytical technique of HPLC coupled with other detectors have been extensively used for the qualitative and quantitative analysis of HMP. HPLC coupled to mass spectrometry is also becoming very popular in the qualitative and quantitative analysis of HMP more especially in the presence of the various types of soft ionization technique. The development of the different types of mass analysers such as Quadruple (Q), time of flight (TOF), and Ion trap (IT) also makes the qualitative and quantitative analysis of complex HMP applicable.

The qualitative analyses of HMP are generally used for the identification of unknown compounds by comparing them with standard ones [104]. However, despite the presence of various powerful analytical tools, the confirmation of chemical component contained in the HMP is still a challenge. This is due to the fact that most of the components in HMP receipts are limited in target compound and the identification techniques rely on the authentic standard [206]. HPLC analysis has been widely applied for the qualitative analysis of HMPs such as* Brucea javanica* [93],* Scutellaria baicalensis* Georgi [103],* Dalbergia odorifera* [104], and* Panax notoginseng* [102].

After the qualitative analysis, next is the quantitative analysis and this is very crucial as it is the prerequisite for the quality control of the HMP. A number of quantitative analysis methods develop using HPLC are simple, stable, and durable [207]. The coupling of the HPLC with different types of detectors such as the UV, ELSD, and MS is frequently used in the quantitative analysis of HMP. The UV detectors are the most frequently used detectors in the quantification of HMPs despite the fact that they have a poor response in compounds with few or no chromatophores. Many kinds of components present in HMP were quantified using this detection technique which includes Yiqing capsule [208] and Qingfu Guanjieshu (QFGJS) capsule [167]. However, these obstacles are usually overcome through the use of ELSD which can detect and quantify the nonchromophoric compounds present in the HMP. Some of the HMPs quantitatively analysed using the ELSD include* Flos Lonicerae* [209] and* Panax notoginseng* (Burk.) F. Chen [210]. The couplings of HPLC with MS are mostly used in quantitative analysis of HMP due to their high rate of sensitivity and selectivity. When developing HPLC-MS method for the quantification of complex material, soft ionization technique such as the ESI and API, detection mode, and the condition of the mobile phase should be optimized [207]. The development of various types of mass analysers as earlier stated can make the quantitative analysis of HMP practically feasible. The HPLC-MS has been widely applied in the quantitative analysis of several HMPs such as Radix Angelicae Dahuricae [105],* Yucca gloriosa L.* [106], Shenqi Fuzheng injection [107], and* Panax notoginseng* (Sanqi) [102].

Apart from the quantitative and qualitative analysis of the HMP, some semiapproaches have been proposed [206]. This approach gives room for the simultaneous determination of a multiple active compound based on the presence of only one or several standards. For example, Gao et al. [108] selected rhizome and used a single standard emodin as both internal and external standard to simultaneously determine other six anthraquinones in rhubarb. The result showed that there was no significant difference between the results obtained using the external standard. The result shows that this alternative method can be used without standard. Simultaneous absolute or semiquantification of multiple components has been widely applied for the quantitative analysis of various HMPs such as* Brucea javanica* [110],* Triticum durum* [111], and* Fritillaria* [109]. The examples of some selected qualitative, quantitative, and semiquantitative analysis of HMP are shown in Table 3.

### 2.5. Data Analysis

Most of the data analyses from HMP are carried out using PC-based software such as the Chromeleon for Dionex which normally comes with the HPLC system and can be used for quality control and authentication purposes. In case of the chromatogram, the data may be composed of different components such as peak area, peak height, and retention time while for spectroscopic (MS or NMR) techniques; the data are obtained in the form of absorbance or transmittance absorbance. These data are analysed for the quantification and identification of separated component of the HMP. These data are analysed and obtained from HPLC fingerprint analysis of the HMP.

Other than the above technique, chemometrics and principal component analysis (PCA) are also techniques used to verify and distinguish the similarity or dissimilar structure of obtained data in the quality control and authentication of the HMP. The chemometrics analysis is composed of group of methods which include basic statistics, mathematics, signal processing, detection, and other methods in order to retrieve more information from the chromatographic data [29]. The chemometrics analysing technique was first coined by Svante Wold in 1971 and is a simple application that integrates mathematics and statistical technique to provide more information from the various spectroscopy and chemical measurement data [211]. However, with the massive progress observed in both electronics and computational technique, chemometrics has become one of the most important and frequently used tools for the analysis of HMP as it helps to provide useful information from the original statistics.

The chemometrics techniques are usually classified into two categories: pattern recognition methods (unsupervised and supervised) and multivariate calibration for qualitative and quantitative evaluation, respectively [212]. The principal component analysis (PCA) and clustering analysis (CA) are the most commonly unsupervised patterns of recognition technique used in the analysis of HMP. The PCA portrays the original measurement by discovering the dominant factors while excluding the relevant interference factors, thereby allowing a more accurate estimate [211]. The PCA technique is also the most commonly applied fingerprint used for handling multivariate data without prior knowledge about the sample under investigation [29].

In terms of the cluster analysis, the objects are grouped based on their chemical characteristics, that is, similar objects cluster (group) more together (forming a branch structure called dendrograms) to each other than those of the other group. The clustering technique is generally divided into two subtypes which include the hierarchical clustering analysis (HCA) and nonhierarchical one. The hierarchical clustering analysis (HCA) is the most popular clustering technique used in the quality evaluation of HMP [211]. The flexibility to alter the similarity measurement criterion applied the linkage method to suit different applications and all the results are always on the original scale of the data, which are the main advantages of the HCA [212]. The combination of PCA and CA has been widely used in identification and authentication of medicinal plants due to the fact that the former can directly reflect the differences between samples whereas the latter can classify objects based on their quantitative characteristics [212].

Peng et al. [113] analysed the fingerprint of* Artemisia selengensis* Turcz. using HPLC-PAD. The samples were analysed using PCA, HCA, and SA. The results from each method used produce different properties of the data matrix from the different* Artemisia selengensis *Turcz. sample type. The study provides important information about the matching and discrimination of the fingerprint. Guo et al. [118] developed HPLC-DAD to simultaneously determine 10 triterpenoid acids from the roots of* Ziziphus jujube*. HCA and PCA were used to differentiate and classify the sample content of the 10 triterpenoid. The PCA technique was also used to analyse HPLC data generated for the authentication of the flavonoid content of different basil cultivars [112, 117].

The linear discriminant analysis (LDA),* k*-nearest neighbour (*k*-NN), soft independent modelling of class analogy (SIMCA), artificial neural network (ANN) partial least squares–discriminant analysis (PLSDA), and orthogonal projections to latent structures–discriminant analysis (OPLS-DA) are the most intensively used supervised pattern recognition in the analysis of HMP [213]. However, before embarking on chemometrics technique analysis, the data are usually pretreated and the goal is to remove unwanted components or unclear interference which can cause overlap of peaks and shifted baseline on the HPLC chromatogram [180]. Examples of some chemometric methods used for the analysis of HMP are shown in Table 4.

### 2.6. Limitation of HPLC in the Authentication of Herbal Medicinal Products

The HPLC chemical fingerprint is the technique that is mostly used as a chemical method for authentication of HMP [214]. However, despite its wide acceptance, it has a number of limitations. One of the crucial limitations of this method is the unavailability of many biologically active compounds (reference standard), and even if present, they are often expensive to purchase [215].

The use of HPLC analysis for authentication of HMPs may also be compromised due to the fact that their test results can be significantly influenced by many factors such as variation in climate, phenotype, manufacturing process, and storage condition and even variation in the type of plant tissue used [171, 180, 216–218]. The authentication of the HMP using HPLC is also affected by age and cultivation time. A study reported by Zhang et al. [219] showed that the best harvesting time for shihu ginseng is the fifth year of cultivation because of the high content of ginsenosides in the fifth year. Therefore, optimization of the numerous variables to ensure reproducibility in the chromatography's fingerprint is always difficult.

The inability of HPLC alone to remarkably identify closely related species that shared similar morphological and chemical properties is one of the most concerning shortcomings of this technique [27]. HPLC can only produce indirect evidence of fraud as it lacks the capability of determining the identity of the given species. Certainly, it is very clear that standardization of HMP to comply with international standard is of enormous importance [16]. However, this process is inherently difficult to achieve with chromatography's fingerprint because there is no universally accepted industrial standard [180].

## 3. DNA Barcoding

DNA barcoding is a novel technique which was first coined by Canadian zoologist Paul Herbert of the University of Guelph in 2003, and it identifies closely related allies of lepidopterans using short DNA sequences from the standard part of the genome [44]. He recommended the use of a partial region of mitochondrial cytochrome c oxidase, which is made up of approximately 650 bp for animal identification as the region is sufficient enough to generate the DNA barcode. The mitochondrial region is suitable for DNA barcode as it fulfilled the basic criteria necessary for species identification. The three basic criteria include (i) universality so that it can be easier to be amplified, (ii) high discriminatory power or specification so as to distinguish it from closely related species, and finally (iii) high sequence quality for easy identification [220]. However, this makes the mitochondrial region of the plants a poor choice for species identification due to the fact that their genes evolved slowly and have a low rate of species discrimination which makes it not suitable for DNA barcoding [45, 221].

For that reason, attention moved to chloroplast and nuclear region in order to find which one among them can serve as a standard barcode for species identification in the plant. After several assessments and exploitation of these loci regions, it was later concluded that multilocus barcode is a requisite for plant barcoding as most of them have one or more limitation; as such their combination was suggested [45]. For example, the two chloroplast regions of maturase K (*mat*K) and large subunit of ribulose-biphosphate carboxylase (*rbc*L) have been found to be suitable barcode region when combined together, a situation that makes the Consortium for the Barcode of Life (CBOL) plant working group recommend them as the universal barcode [222].

The* rbc*L barcode region, which consists of approximately 1430 bp, is easy to be amplified and has the ability to generate good quality sequence [223]. The limitation of using this region is its length, which in some cases needs to be entirely sequenced for species discrimination [224]. However, other* rbc*L primers such as 1F/724R which has been proposed as a universal primer in gymnosperm due to its high rate of universality [225] can be used to amplify part or half of the* rbc*L gene. The length region of the* rbc*L has made it have poor discriminatory power more especially at the species level [226, 227]. The long size and limiting factor of* rbc*L region makes it not suitable for species identification because an ideal DNA barcode region should be short to be amplified from degraded DNA [228], in terms of the latter universal barcode region, which has the closest analogue to the COI animal barcode [229] that consists of 841 bp at the centre of the gene (Staats et al., 2016b). The chloroplastic* mat*K region is one of the most rapidly evolving genes with suitable length and high level of discrimination among angiosperm [230]. The difficulty in the amplification of* mat*K more especially in nonangiosperm is one of the limitations of using this barcode region and this can be linked as a result of its insufficient universal primers [231, 232]. The combination of these two loci is found suitable for DNA barcode due to the high amplification rate of the* rbc*L region and discriminatory power of* mat*K region [233].

However, despite the fact that the combination of* rbc*L +* mat*K offers slightly higher identification efficiency than other combinations, their ability to discriminate between closely related species is lower than that of COI in animals [224, 234]. In addition to that, the combined barcode also has a low PCR efficiency of* matK* and this explains the fact that the* rbc*L +* mat*K barcode still failed to meet the original goal of universal barcode [224]. Increase in analytical difficulties is always an issue in combining barcodes compared to single locus-marker, more especially when one of the target regions failed to be amplified.

These developments opted China plant Barcode of Life (BOL) working group to propose the use of another nuclear region (ITS) in addition to the standard barcode [45]. They argued that the ITS regions have more discriminatory power than the plastid barcodes [233] despite the limitations associated with the regions such as difficulties of amplification, sequencing, and incomplete concerted evolution [235]. In order to resolve the difficulties in amplification and sequencing of the ITS region, they suggested the use of the short ITS2 region as a backup because of the presence of it conserve sequences [224]. The ITS region in addition to another noncoding chloroplast region (*psb*A-*trn*H) is the most widely used supplementary loci for species identification [236].

Subsequently, the options of using either three or two barcodes were later discussed in the fourth international Barcode of Life conference held in Australia. It was concluded that the two-barcode region was preferred in order to avoid the low cost of sequencing [237]. Therefore, the use of* rbc*L and* mat*K DNA barcodes regions as standard barcodes region together with ITS and* psb*A*-trn*H regions serving as complementary barcodes has helped tremendously in identifying herbal medicines and their adulterants [220]. Further standard DNA barcodes regions which have been used in authentication of HMP include* trn*L-*trn*F [125] and* rpo*C1 [146].

The chloroplasts are very important region used for the identification of HMPs. This is due to the fact that this region contains highly conserved and variable genes which are fundamental and informative to plants over broad time scale. Nock et al. [238] use complete chloroplast genome to identify species and this has become a universal method used for single locus barcode identification, taxonomy, and Phylogenetic analysis of plant species. The authors felt that the massive parallel sequencing (MPS) which they used can significantly improve the ability of distinguishing between and identifying different species, and it is simple and cost-effective.

### 3.1. DNA Barcoding for Identification of HMPs

The process of identification of species using DNA barcoding can be achieved by building the DNA barcode library of known species and matching or assigning the unknown barcode sequence against the barcode library [239]. In terms of HMPs, these processes can be achieved in five basic steps which are of enormous importance as error or misinterpretation can arise within each step. The steps followed include (1) homogenisation of the medicinal herbal product to ground powder, (2) extraction of the genomic DNA, (3) amplification of the specific DNA barcodes region using PCR, (4) sequencing of the amplified region, and finally (5) identification of the unknown sequence against known standard reference material [49].

#### 3.1.1. DNA Extraction

Extraction of DNA of high quality is the most crucial step and prerequisite for proper identification of HMPs. Extraction of any cellular components usually involves three central steps: (1) disruption of the cell walls, (2) removal of insoluble particulates from DNA, and finally (3) DNA precipitation with integrity maintenance [240]. Due to the physical nature of the plant cell wall, mechanical disruption such as the use of pestle and mortar, glass beads, steel or glass rod, and enzymatic digestion must occur before extraction [241]. A physical disruption method using liquid nitrogen is more preferred as it will help to prevent cross contamination more especially when multiple samples are used [242]. The use of chemical disruptors is also convenient as it can bypass the possibility of DNA shearing due to the mechanical force applied [243]. The prime limitation of using chemical disruptors is that they are expensive [240].

Like any other type of molecular technique, DNA barcoding relies on the availability of high quality DNA for species identification [50]. Therefore, any extraction method that will produce higher DNA yield and quality from HMP will be of enormous importance. This is due to the fact that HMP undergoes a series of heavy processing activities such as drying and stewing, and this may result in the fragmentation or degradation of the DNA to be used for DNA barcoding. In addition, most HMPs contain high amount of secondary metabolites derived from raw materials (e.g., polysaccharides, polyphenolic compounds, and pigments) within various tissues and organs of the plants [244, 245]. The presence of these metabolites may prove to be the main cause for extraction of low quality DNA by binding with it and precipitating along with it [246].

The different types of extraction methods which can be used for extraction of DNA of high quality from HMP are now available; this can be based on conventionally developed protocols or commercially available kits (Table 5). The use of commercial kits for extraction of DNA from HMP is sometimes necessary as it used to extract DNA of high purity. Another advantage of using commercially available kits is the minimal requirement for laboratory equipment; as such, it can easily be implemented in any laboratory. The majority of the commercially available kits used for DNA extraction in HMP use silica binding extraction methods. As such, the affinity of the column through which the membrane binds with the DNA is very important. The prime limitation with commercially available kit is that, it may not be available for low funded laboratory or routinely used for extraction of a large number of samples [247]. Furthermore, it does not provide room for researchers to modify the standard protocol as the kits provided are limited.

However, other in-house DNA extraction protocols such as Cetyltrimethylammonium bromide (CTAB) extraction buffer utilize salting out precipitation method. This extraction method has been shown to be cost-effective compared to commercially available kits although it is time consuming [248, 249]. The combined use of two extraction protocols such as CTAB with silica binding or resin has showed a promising result in a wide range of plant and plant derived products [250–252].

However, like the CTAB method, sodium dodecyl sulfate (SDS) which is an anionic detergent is also used in the extraction of DNA from plant derived products. The SDS is used in the disruption of the cell membrane to release the DNA into the extraction buffer after breaking or digesting away the cell wall and nuclear membrane [253]. The use of SDS in the extraction of DNA developed by Edwards et al. [134] is a simpler, faster, and inexpensive technique. Besides the digesting of the cell wall and nuclear membrane, SDS together with EDTA present in the extraction buffer protects the DNA from endogenous nucleases by inhibiting their activities [254]. The activities of endonucleases are inhibited in the presence of EDTA, which immediately chelates with divalent ions (mg^+2^, Ca^+2^, and Mn^+2^), the necessary cofactors required for the enzyme structure and function [255].

#### 3.1.2. Amplification of DNA Barcoding Region

Amplification of the DNA barcode region is one of the most important steps for species identification and authenticity and this is solely dependent on the quality of the DNA template. The relatively long gene sequence which is required for proper DNA barcoding (ranging from 500 to 1000 bp) [49] is mostly not found in HMPs as they get fragmented into pieces. Fragmentation of DNA usually occurs as a result of a process in which the herbal starting material may have been dried in the sun or heavily processed to the extent that the DNA to be isolated is degraded [256]. Fragmentation or degradation of DNA molecules extracted from the herbal medicinal material is the primary cause of PCR failure [48].

Therefore, to overcome the problem of PCR amplification, appropriate PCR primers (e.g., primers with high affinity) such as the novel minibarcode will be of enormous importance as they have high amplification success compared to the full length barcode in degraded form of DNA [257]. Särkinen et al. [252] who were working on degraded form DNA (herbarium DNA) reported that there is a negative correlation between amplicon size and PCR success, indicating that smaller fragments are easy to be amplified. The barcode region to be used in authenticating HMPs should not be large as most of the failure of the PCR amplification from degraded DNA samples is frequently reported when the amplicons are greater than 200 bp [48].

#### 3.1.3. Sequencing of the Amplified Region

Generation of sequence of high quality is one of the most important criteria for species identification and authentication. The level of DNA degradation at which useful sequence can be generated for species identification varies with the type of methods used; for instance, high-throughput sequencing approach can be effective with a small fragment size (around 50–400 bp) [50]. However, in other sequencing techniques, such as Sanger, the PCR needed to amplify the DNA requires the fragment of at least the size of amplified fragment [50]. So, also, higher chances of successful amplification of the target barcode marker are possible with less fragmented total DNA yield. Next-Generation Sequencing (NGS) technology has several key advantages over the conventional Sanger sequencing [258]; as such, it will offer a tremendous impact in the authentication of the herbal products. Some of the advantages of NGS technique include (1) high-throughput, (2) massive parallelization of sequencing reaction, (3) low cost and operation difficulties, (4) superior sensitivity, and (5) also the ability to be used to sequence large and more complex sequences [258, 259]. This in turns makes NGS the most preferred method for analysing samples with various degrees of DNA degradation.

#### 3.1.4. Reference Sequence Data

The identification and authentication of herbal medicinal plant by DNA barcoding solely depend on the availability of reference sequence data. This is very important because, in the absence of reliable reference sequence, identification of unknown samples may not be accurate [49]. The sequence data that are used for this identification are currently deposited in the public library and are free to access. The most common ones among them include the following.

*(1) BOLD (The Barcode of Life Data System).* This is an information workbench which was created and maintained by the Guelph in Canada. Presently, it contains over 370,000 plant barcodes representing over 58,510 species of plant [50] and this includes vouchers, images, and maps. BOLD also partners with other public reference libraries which include CBOL [Consortium for the Barcode of Life], IBOL [International Barcode of Life (http://www.ibol.org)], GBIF [Global Biodiversity Information Facility (http://www.gbif.org)], and NCBI GenBank [National Centre for Biotechnology Information (http://www.ncbi.nlm.nih.gov)]. One limitation of the BOLD database is that many medicinal plants are missing. So, also, various important barcodes regions such as ITS2 do not have complete coverage.

*(2) NCBI GenBank.* GenBank is probably one of the biggest and most important publicly available online databases built by NCBI. GenBank is one of the most frequently used databases of genetic information [260]; as such, the assembled sequence in its database has exceeded 1 Terabase in October, 2014 [50]. GenBank contains BLAST (basic local alignment search tool) algorithm [261] through which an unknown (query) DNA sequence can be rapidly and accurately compared with known (reference) sequence.

*(3) MMDBD (Medicinal Materials DNA Barcode Database).* This is a database which mostly contained DNA sequence and information on medicinal plants listed in both Chinese and American pharmacopeia. Presently, this database contains over 15,375 sequences representing 1660 species of medicinal material and also contains multiple regions, including 4 nuclear and mitochondria regions each and 7 chloroplast regions [50].

### 3.2. Limitation of DNA Barcoding in the Authentication of HMPs

Basically, there are various limitations of DNA barcoding in the authentication of HMP and the most crucial among them is the low quality extraction of DNA template. Different drying methods and series of heavily processing activities in addition to the presence of secondary metabolite in the plants might result in the extraction of degraded or fragmented form of DNA, thus resulting in broken strands DNA [262]. If one of these breakages occurs at the primer annealing site, amplification will not occur and the reaction will fail. Fragmented or degraded form of DNA is not suitable for DNA barcoding [263]. This is due to the fact that the extraction of DNA of high quality is the prerequisite for the identification and authentication of HMPs. Useful sequence which can be generated for species identification and authentication in DNA barcoding varies with the level of DNA degradation and type of sequencing methods used [50]. High-throughput sequencing methods can work effectively with fragment size of 50–400 bp while other approaches such as Sanger will require fragment of at least the size of the amplified fragment (>500 bp), but high chances of amplification success of the target DNA barcodes are found in less fragmented extracted DNA.

The Sanger sequencing always required pure PCR product for effective and reliable sequencing; that is, PCR product from multiple or complex mixture is not suitable for sequencing using Sanger sequencing [50]. This is due to its inability to resolve mixed signals from such kind of samples [258]. However, such limitation can be overcome by involving the use of next-generation sequencing (NGS) which has several advantages over the Sanger sequencing. Yielding millions of DNA reads in a small period of time, massive parallelization of sequencing reaction, better sensitivity, and clonal separation of templates are among the factors that make NGS technology perform effectively compared to the Sanger sequencing (Staats et al., 2016a). The coupling of DNA barcoding together with NGS is referred to as metabarcoding [264] and has been applied in the diverse identification of species. However, only few publications demonstrated the power of metabarcoding in the authentication of complex HMPs. Coghlan et al. [265] and Cheng et al. [266] performed a DNA metabarcoding analysis of different types of complex traditional Chinese medicine (TCM) and both concluded that metabarcoding can serve as a prospective way to authenticate complex HMPs. Ivanova et al. [258] also recently reported that DNA metabarcoding can be reliably used in the authentication of complex HMPs. They further emphasized that that Sanger sequencing should not be used as they lack the ability to resolve mixed signals sample containing multiple species.

The processes by which standardized extracts are produced may lead to the degradation or removal of plant DNA from the raw material, therefore making DNA barcoding unfeasible for the authentication of such HMPs [50, 258]. This is due to the fact that the process involves multiple steps [171]. In addition, some artificial adulterants used do not have DNA, thus making the DNA barcoding unfeasible for verifying their identity in any given HMP sample [27]. Solid state fermentation, which is one of the strategies used to improve the yield of active compounds [267, 268] can also result in the extraction of low quality DNA as they can be partially or fully degraded [49]. The presence of foreign or contaminating DNA from microbial organisms such as fungi which may accumulate on improperly stored plant material can be amplified particularly when using nuclear fragment (ITS or ITS2) primers [49, 269, 270]. This situation can result in the generation of PCR product which might be difficult to analyse [269].

Another disadvantage of using DNA barcoding in authenticating the HMP is that it cannot differentiate tissue within the same plant; that is, it cannot identify the plant part used to make the HMP. For example,* Eurycoma longifolia* is a famous medicinal plant in Malaysia solely known for its energetic and aphrodisiac property. Most of the chemical compounds responsible for this therapeutic effect are present on the root part; as such, most of the research on this plant is conducted on the root part [271]. Substitution within the same species part of this plant (e.g., substituting the root part with leaf part) cannot be detected using DNA barcoding. So, also, despite the effectiveness of DNA barcoding in identifying various adulterants used in the production of HMP, it lacks the ability of providing information regarding the presence and concentration of active ingredients responsible for their therapeutic properties [27]. This explains the fact that DNA barcoding cannot provide information on whether a particular HMP has met the pharmacopeia standard as this is necessary for overall quality control. Thus, DNA barcoding can only determine the authenticity of HMPs but cannot be used to evaluate their quality.

The affinity of the primer is also very important as this can yield a false negative result [50]. If the products that need to be authenticated contain a DNA from another plant which has great affinity with the barcode primer, it can therefore be preferentially amplified, thus leading to a false amplification and sequencing. This situation mostly occurs in target species whose DNA is degraded into short pieces [258]. The relatively long gene region of 500–1000 bases that is required for DNA barcoding is not mostly intact in HMPs due to the fact their DNA is mostly degraded or fragmented into short pieces as a result of a series of processing activities which they underwent [49]. Therefore, it is recommended that shorter DNA barcodes region, so-called minibarcode, of less than 150 bp should be created as this will help to overcome the problem of PCR amplification [50]. Failure of PCR amplification has been frequently reported when the amplicon size is greater than 200 bp [48, 142, 272, 273]. Minibarcode in general has a high amplification success in degraded sample compared to standard or full length DNA barcode [252, 257].

Sequencing is also one of the main limitations of DNA barcoding as different sequencing methods have different limitations. Low throughput and the requirement of high concentration of DNA amplicon template of about 100–500 ng are the inherent limitation of using this approach so as to avoid bias and error [274]. The Sanger sequencing is the most commonly used method in the authentication of HMP to date [148, 275]. This approach is mostly affected by primer bias, even if serial dilution and multiple extraction methods are used [258]. The effects of PCR bias and amplification can be partly solved by ligation of the PCR fragment into vector and cloned in bacteria or by performing parallel PCR [50].

DNA barcoding cannot identify adulterants present in HMP that do not contain DNA [27]. For example, the use of hazardous sulphur fumigated processing which has been reported to be used by herbal farmers or wholesalers in order to extend the storage time and prevent insect infestation [14] cannot sufficiently confirm using DNA barcoding. The HMPs treated with sulphur, which appeared very clean and bright in colour, do not only destroy the chemical or biological properties of the HMP but also have toxic effects as the sulphur is realised to the environment [276].

The use of public database such as the GenBank for species identification is another limitation of using DNA barcoding for the authentication of HMPs. This is due to the fact that the reference sequences of some medicinal plants are insufficient or not available and this makes the authentication process difficult [277]. The GenBank mostly provides useful information when doing a preliminary research or when dealing with limited resources. The scarcity of the complete sequence in the GenBank is another limitation when using GenBank for species identification. The partial sequences from a less variable portion of a gene may result in an inaccurate identification of the query sequence [277]. Another potential problem of using GenBank database is the wrong submission of sequences which can lead to inaccurate identification of the query sequence [278, 279]. The provision of barcodes for many medicinal plant species by GenBank and MMDBD but without information about the voucher specimen will likely result in some misidentification [277, 280].

Ensuring of unequivocal species identification of raw materials that will be used by herbal industries in the manufacturing of HMPs is always a challenge (Palhares et al., 2015a). This is due to the fact that the various conventional methods of identifying herbal medicinal plant such as macroscopic and microscopic methods and the organoleptic method cannot identify the species in a processed product as both methods require human expertise [45]. So, also, the use of chromatography (e.g., HPLC) or molecular (e.g., DNA barcoding) fingerprints alone may not provide the detailed information about the composition of the herbal medicinal plants despite their wide acceptance. This is because each of the methods has its own limitation. The combination of both methods can help to provide detailed knowledge about the main composition of the HMPs. Some studies suggested that the DNA barcoding should be used in a complementary manner with chemical analyses for species analysis as these will tremendously help to ascertain their efficacy and safety as these are very important for quality control (Table 6).

Li et al. [125] used DNA barcoding, HPLC, TLC, and cytotoxicity assay to authenticate 10 samples retailed as Baiying and Xungufeng HMPs. Baiying is a natural product used for the treatment of cancer, derived from* Solanum lyratum*. Baiying has a common substitute present in the market called Xungufeng, a carcinogenic aristolochic acid-containing herb derived from* Aristolochia mollissima* Thunb.

A total of 30 sequences were generated using 5 DNA barcode regions (ITS,* mat*K,* rbc*L,* trn*H-*psb*A, and* trn*L-*trn*F) to differentiate these two plants. The five barcode regions were also applied to authenticate the 10 samples of Baiying and Xungufeng HMPs. The sequence result of all the five barcodes regions revealed that two samples of Baiying were derived from* Solanum lyratum* while the remaining three were substituted with* Aristochia mollissima* Thunb which contained the carcinogenic aristolochic acid. So also, for the HMP labelled as Xungufeng, three samples were derived from* Aristochia mollissima* Thunb while two were substituted with* Solanum lyratum*. Further authentication of the HMPs using HPLC and TLC showed that the results are in agreement with that obtained from DNA barcoding analysis.

The most recent of such studies is that reported by Palhares et al. [143]. They analysed 257 samples which consist of dried leaves, flowers, and roots from 8 distinct species of medicinal plants sold in Brazil using three barcoding (*rbc*L,* mat*K, and ITS2) regions. The result revealed that 42% of the samples belong to the correct genus while the level of substitution was high, being around 71%. Qualitative and quantitative analysis revealed that the correct ones are sometimes sold, but the chemical compounds are not present. The result from this study shows that both of the techniques have their own limitation, but, by combining them together, more information can be obtained.

## 4. Conclusion

DNA barcoding is a reliable and suitable technique used for the identification of HMPs and for the determination of various adulterants under specific conditions, even if they existed in a processed products form such as capsules, powder, tablets, or dried form. However, in the absence of any conditions such as good quality genomic DNA, good primer affinity for successful amplification, good sequencing methods, and authentic reference library, identification using DNA barcoding can be challenging. Another limitation of using DNA barcoding for identification of HMPs is the inability to provide information about the presence and concentration of a specific active ingredient so as to determine whether it has met the pharmacopeia standard. This shows that DNA barcoding can only authenticate HMPs but cannot evaluate their quality. On the other hand, chromatography fingerprint can be used to evaluate the quality of HMPs due to the fact that it can provide information about the presence and concentration of a specific bioactive compound. Like the DNA barcoding, chromatography fingerprint may also be affected by some factors such as physiological and storage conditions. Another limitation of using chromatography fingerprint in the authentication of HMP is that it can only produce indirect evidence of fraud as it cannot determine the identity of the given species.

Taking account of all the advantages and inherent limitation of DNA barcoding and chromatography fingerprint analysis, the combination of the two techniques could be an added advantage for comprehensive quality assessment of HMPs as these are necessary for quality control. Therefore, with the global increase in the demand and consumption of HMP, a combination of these great techniques to work in synergy with each other will help to check their quality as this is critical for efficacy and safety. The information will in turn help to change the consumer's perception that everything natural is safe as they tend to have relatively poor understanding of their safety. They will tend to understand that some of the HMPs are not effective and some can cause serious health problems. The information may also help to increase the consumers' overall confidence in the consumption of HMP more especially if they are of quality standard.

## Figures and Tables

**Table 1:** Examples of polyherbal HMPs.

Polyherbal product	Medicinal value	Composition	References
Xue-Fu-Zhu-Yu decoction	Treatment of atherosclerosis and coronary heart disease, lowering the level of cholesterol in serum	*Paeonia lactiflora*, *Ligusticum chuanxiong*, *Citrus aurantium Carthamus tinctorius*, *Prunus persica*,and *Bupleurum falcatum*	Zhang and Cheng [65]

Ayurved Siriraj Prasachandaeng	Antipyretic drug	*Myristica fragrans, Citrus aurantifolia, Bouea macrophylla, Knema globularia, Caesalpinia sappan, Conioselinum univitatum, Dracaena loureiri, Kaempferia galanga, Mesua ferrea, Jasminum sambac, and Mammea siamensis Nelumbo nucifera*	Akarasereenont et al. [66]

Ge-Gen Decoction	Treatment of common cold, fever, and influenza. It is also used for the treatment of different diseases, such as cervical spondylosis and primary dysmenorrhea	Pueraria Radix (Gegen), Ephedrae Herba (Mahuang), Cinnamomi Ramulus (Guizhi), Glycyrrhizae Radix et Rhizoma Preparata (prepared Gancao), Paeoniae Radix Alba Preparata (prepared Shaoyao), Zingiberis Rhizoma Recens (Shengjiang), and Jujubae Fructus (Dazao).	Yan et al. [67]

Danggui-Shaoyao-San (DSS)	Treatment of gynaecological disorder such as amenorrhea and dysmenorrhea	Radix *Paeoniae alba*, Radix *Angelica sinensis*, Rhizoma *Chuanxiong, Poria cocos*, Rhizoma *Atractylodis macrocephalae,* and Rhizoma *Alismatis*	Chen et al. [68]

Shrishadi	To treat infectious respiratory disorders	*Albizia lebbeck*, Cyprus rotan, and *Solanum xanthocarpum*	Kajaria et al. [69]

Hongdoushan capsule	Treatment of ovarian and breast cancers	*Glycyrrhiza uralensis Fisch., Panax Ginseng, Taxus chinensis var. mairei*	Zhu et al. [70]

**Table 2:** Example of some detectors used for the detection of HMPs.

Types of HMPs	Medicinal value	Column	Detector	Chromatographic condition	References
Valerian	Neuralgia, epilepsy, and relieving digestive	VP-ODS C18 column (4.6 × 250 mm, 5 *μ*m),	HPLC-UV	^a^Acetonitrile; 0.8 mL/min isocratically; ^d^10 *μ*L	Ghafari et al. [71]

Vidanga	Anthelmintic, carminative, and stimulant	Chromatopak Peerless basic C18 column (4.6 × 250 mm, 5 *μ*m)	HPLC-PDA	^a^MeOH-phosphate; ^b^1.4 mL/min isocratically; ^c^25°C, ^d^20 *μ*L	Sudani et al. [72]

Radix Isatidis *(Isatis indigotica)*	Influenza, epidemic hepatitis, and epidemic encephalitis B	ODS-3 Inertsil column (25 cm × 4.6 mm i.d., 5 *μ*m)	HPLC-DAD	^a^Water-acetonitrile; ^b^1 mL/min gradient; ^c^35°C; ^d^10 *μ*L.	Zou et al. [73]

Buyang Huanwu decoction	Promoting blood flow, treatment of cerebrovascular diseases	Zorbax SB-C18 column (4.6 × 50 mm, 1.8 *μ*m)	HPLC-DAD-TOF/MS	^a^0.3% formic acid water- acetonitrile; ^b^0.8 mL/min gradient; ^c^25°C; ^d^1 *μ*L.	Liu et al. [74]

*Ginseng*	Antidiabetic, anti-inflammatory, and antitumour activities	C18 column (250 mm × 4.6 mm i.d., 5 *μ*m)	HPLC-ELSD	^a^Acetonitrile-water-acetic acid (10 : 85 : 5, v/v/v) and Acetonitrile-water (80 : 20, v/v); ^b^1.5 mL/min gradient; ^c^40°C; ^d^10 *μ*L.	Sun et al. [75]

Ge-Gen decoction	Treatment of common cold, fever, and influenza	Agela Venusil MP-C18 column (4.6 × 250 mm i.d., 5 *μ*m)	HPLC-Q-TOF-MS/MS	^a^0.1% formic acid-water-acetonitrile; ^b^1.0 mL/min gradient; ^c^30°C; ^d^10 *μ*L	Yan et al. [67]

*Radix Paeoniae Rubra*	Anti-inflammatory, increasing coronary blood flow	Waters Symmetry C18 column (250 mm × 4.6 mm i.d., 5 *μ*m)	HPLC-PDA	^a^Acetonitrile-phosphoric acid; ^b^0.8 mL/min gradient; ^c^25°C; ^d^20 *μ*L	Xu et al. [76]

*Cimicifuga racemosa*	Antipyretic and anti-inflammatory activities	C-18 column (5 *µ*m, 120 Å, 4.6 mm × 250 mm)	HPLC–PDA–ELSD	^a^Acetonitrile-water; ^b^1.6 mL/min gradient; ^c^43°C; ^d^20 *μ*L	Li et al. [77]

Renshen *(Panax ginseng)*	Treatment of cardiovascular diseases, cancer	A CSH C18 column (2.1 × 100 mm, 1.7 *μ*m)	HPLC/QTOF-MS^E^	CH_3_CN-H_2_O; ^b^0.3 mL/min gradient; ^c^25°C; ^d^2 *μ*L	Qiu et al. [78]

*Glycyrrhiza glabra L*	Anti-inflammatory, antiviral, antiallergy, and antiulcer properties	C18-HL (150 × 100 mm, 10 *μ*m Hichrom Ltd)	HPLC-PDA	0.1% TFA in water-0.1% of TFA in MeOH; 3.00 mL/min gradient; ^c^25–28°C; ^d^100 *μ*L	Basar et al. [79]

Sanqi *(Panax notoginseng)*	Hemostatic and cardiovascular properties	Waters Symmetry C18 column (250 mm × 4.6 mm i.d., 5 *μ*m)	HPLC-UV	^a^Water-acetonitrile; ^b^1.0 mL/min gradient; ^c^35°C; ^d^5 *μ*L	Lau et al. [80]

Renshen *(Panax ginseng)*	Treatment of cardiovascular diseases, cancer	Waters ODS C18 column (150 × 2.1 mm i.d.; 5 *µ*m)	HPLC-APCI/MS	^a^Acetonitrile-water (33 : 67, v/v); ^b^0.2 mL/min isocratically; ^c^35°C	Ma et al. [81]

*Evodia rutaecarpa*	Anti-inflammatory and antibacterial effects	Zorbax SB-C18 column (250 mm × 4.6 mm i.d., 5 *μ*m)	HPLC-DAD	^a^Acetonitrile-water; ^b^1.0 mL/min gradient; ^c^25°C; ^d^10 *μ*L.	Zhao et al. [82]

*Fritillaria pallidiflora*	Treatment of cough	Kromasil C18 column (200 × 4.6 mm i.d.)	HPLC-ELSD	^a^Acetonitrile-water-diethylamine (70 : 30 : 0.1, v/v); 1 mL/min isocratically; ^c^88°C, ^d^20 *μ*L	Li et al. [83]

Hongdoushan capsule	Treatment of ovarian and breast cancers	Shimadzu C18 column (4.6 × 250 mm, 5 *μ*m)	HPLC-PDA	^a^Acetonitrile-water; ^b^0.8 mL/min gradient; ^c^25°C; ^d^5 *μ*L	Zhu et al. [70]

*Panax quinquefolium*	Improving cardiovascular activity, insomnia	Waters Spherisorb S3 ODS2 column (150 · 2.0 mm)	HPLC-APCI-MS	^a^Acetonitrile : water (24 : 76, v/v); ^b^0.2 mL/min isocratically; ^c^35°C	Ma et al. [84]

*Coptidis rhizome* (Huanglian)	Suppressing fever, dispelling dampness, and antimicrobial properties	Kromasil C18 analytical column (250 mm × 4.6 mm, 5 *μ*m)	HPLC-ELSD	^a^Acetonitrile : water (30 : 70, v/v) pH was adjusted to 6.0 with 0.2 moL/L trichloroacetic acid; ^b^2.8 L/min; ^c^115°C; ^d^20 *μ*L	Kong et al. [85]

Menoprogen	Treatment of menopause	Kromasil ODS C18 column (4.6 × 250 mm, 5 *μ*m)	HPLC-PDA	0.1% phosphoric acid- 0.1% acetonitrile; ^b^1.0 L/min; ^c^25°C; ^d^10 *μ*L	Wang et al. [86]

*Fructus Aurantii Immaturus*	Dispersing painful abdominal mass	Sepax C18 column (5 *μ*m, 250 mm × 4.6 mm)	HPLC-DAD	^a^Acetonitrile-methanol-polyphosphoric; ^b^0.8 mL/min gradient; ^c^30°C; ^d^20 *μ*L	Xu et al. [87]

Tong-Xie-Yao-Fang	Diarrhoea-predominant irritable bowel syndrome	Kromasil C18 column (250 mm × 6 mm, 5 *μ*m)	HPLC-DAD-ESI-M	^a^Acetonitrile-0.1% formic acid and water; ^b^0.6 mL/min gradient; ^c^35°C; ^d^5*μ*L	Yan et al. [88]

Tulsi *(Ocimum sanctum)*	Carcinogens, chemotherapeutic agents	C18 column (250 × 46 mm^2^, 5 mm; Waters, USA)	HPLC-PDA	^a^Water-acetonitrile; ^b^1.0 mL/min gradient; ^c^40°C;	Chanda et al. [89]

*Panax quinquefolium*	Improving cardiovascular activity, insomnia	Diamonsil C18 column (5 *μ*m, 250 mm × 4.6 mm)	HPLC-UV–vis	^a^Acetonitrile-0.5% of phosphate acid; ^b^1.0 mL/min gradient; ^c^35°C; ^d^20 *μ*L	Zhang et al. [90]

*Eurycoma longifolia J*	Erectile dysfunction, libido, and male infertility	C18 column (2.1 mm × 50 mm, 2.7 *μ*m)	LC-MS/MS	^a^0.1% formic acid in water-0.1% formic acid in acetonitrile/water (90 : 10, v/v); ^b^0.25 mL/min gradient; ^c^40°C; ^d^2 *μ*L	Han et al. [91]

Antike capsule	Antineoplastic property	Zorbax SB-C18 column (4.6 × 250 mm 5 *μ*m)	HPLC-PDA	MeCN and 0.1% HOAc–0.5% KH_2_PO_4_ aqueous solution (adjusted to pH = 2.4 with H3PO_4_); ^b^0.8 mL/min gradient; ^c^30°C; ^d^20 *μ*L	Duan et al. [92]

*Brucea javanica*	Treatment of cancer, amebic dysentery, and malaria	Agilent Eclipse XDB-C18 column (250 mm × 4.6 mm, 5 *μ*m)	HPLC-QTOF/MS	Water-acetonitrile; ^b^1 mL/min gradient; ^c^25°C	Tan et al. [93]

Fufang Zhenzhu Tiaozhi	Dyslipidemia	Dionex Acclaim C18 column (250 mm × 4.6 mm, 5 *μ*m)	HPLC-DAD	^a^Acetonitrile-potassium dihydrogen phosphate solution (40 : 60 v/v) PH adjusted to 3 with 1.7 g/L sodium dodecyl sulfate and phosphoric acid; ^b^1.0 min/mL isocratically; ^c^30°C; ^d^20 *μ*L	Chen et al. [94]

Black cohosh *(Cimicifuga racemosa)*	Antipyretic and anti-inflammatory activities	C-18 column (150 × 4.6 mm, 5 *μ*m)	HPLC-ELSD	^a^Water-acetonitrile-alcohol; ^b^1.0 mL/min gradient; ^c^40°C; ^d^10 *μ*L	Ganzera et al. [95]

*Psoralea corylifolia*	Osteoporosis, osteomalacia	Zorbax SB-C18 column (3.0 × 100 mm, 3.5 *µ*m)	HPLC-DAD-TOFMS-QITMS	^a^0.1% aqueous formic acid-acetonitrile; ^b^0.6 mL/min; gradient; ^c^30°C; ^d^10 *μ*L	Tan et al. [96]

*Cimicifuga foetida* L	Antimicrobial, tranquilizer, and hypotensive treatments	Hypersil, UK, ODS2 column (200 × 4.0 mm i.d., 5 *μ*m)	HPLC-ELSD	^a^Methanol-water; ^b^0.8 mL/min gradient; ^c^30°C; ^d^10 *μ*L	Kong et al. [97]

Xue-Fu-Zhu-Yu decoction	Atherosclerosis and coronary heart disease	Zorbax SB-C18 column (4.6 × 250 mm, 5 *μ*m)	HPLC-ESI-MS	^a^(CH_3_COOH:H_2_O)-(CH_3_OH); ^b^0.5 mL/min gradient; ^c^30°C	Zhang and Cheng [65]

*Curculiginis Rhizoma*	Treatment of pain, inflammation, and immune and hepatic disorders	Altima C18 ODS column (4.6 × 250 mm, 5 *μ*m)	HPLC-DAD	^a^Acetonitrile with 0.02% TFA-water with 0.5% TFA; ^b^1.0 mL/min gradient; ^c^30°C; ^d^10 *μ*L	Bian et al. [98]

Danggui-Shaoyao-San	Dysmenorrhea, amenorrhea	Alltima C18 column (250 mm × 4.6 mm i.d., 5 *μ*m)	HPLC-DAD-ESI-MS	^a^Acetonitrile-water-formic acid; ^b^1 mL/min gradient; ^c^30°C	Chen et al. [68]

Ojeok-san	Primary dysmenorrheal, clastogenicity	Gemini C18 column (4.6 × 250 mm, 5 *μ*m)	HPLC-DAD	^a^0.1% formic acid in water-acetonitrile; ^b^1.0 mL/min gradient; ^c^40°C; ^d^10 *μ*L	Kim et al. [99]

Gui-Zhi-Jia-Shao-Yao-Tang	Effect against peptic ulcer by alleviating gastrospasm	Zorbax SB-C18 Rapid Resolution HT column (4.6 mm × 50 mm, 1.8 *μ*m, Agilent)	LC-MS and NMR	^a^0.05% formic acid in water-acetonitrile; ^b^0.6 mL/min gradient; ^c^30°C; ^d^3 *μ*L	Wang et al. [100]

Renshen *(Panax ginseng)*	Cardiovascular diseases, cancer	Capcell Pak C18 column (250 mm × 4.6 mm i.d., 5 *μ*m)	HPLC-ESI-MS	^a^0.2% acetic acid in water-acetonitrile; ^b^0.5 mL/min gradient; ^c^35°C; ^d^10 *μ*L	Zhang et al. [101]

Sanqi *(Panax notoginseng)*	Hemostatic and cardiovascular properties	Agilent Eclipse XDB-C18 column (250 mm × 4.6 mm, 5 *μ*m)	HPLC-QTOF/M	^a^0.1% aqueous formic acid-acetonitrile containing ^a^0.1% formic acid; ^b^0.8 mL/min gradient; ^c^25°C;	Tan et al. [102]

^a^Mobile phase; ^b^flow rate; ^c^temperature; ^d^injection volume.

HPLC-UV: Ultraviolet-Visible detector; HPLC-DAD: liquid chromatography-diode-array detector; HPLC-PDA: liquid chromatography photodiode-array; HPLC-ELSD: liquid chromatography evaporative light scattering detector; LC-MS: liquid chromatography-mass spectrometric detector; NMR: nuclear magnetic resonance; HPLC-DAD-ESI-MS: liquid chromatography-diode-array detector-electrospray ionization-mass spectrometry; HPLC-APCI-MS: liquid chromatography–atmospheric-pressure chemical ionization-mass spectrometry; HPLC- DAD-TOF/MS: liquid chromatography-diode-array detector-time of flight-mass spectrometry; LC-MS and NMR: liquid chromatography-mass spectrometry-nuclear magnetic resonance; HPLC-QTOF/MS: liquid chromatography-quadrupole time of flight-mass spectrometry; HPLC-ESI-MS: liquid chromatography-electrospray ionization-mass spectrometry; HPLC-DAD-TOFMS-QITMS: liquid chromatography-diode-array detector-time of flight-mass spectrometry-quadrupole ion trap mass spectrometry; LC-MS/MS: liquid chromatography-mass spectrometry/mass spectrometry; HPLC-PDA-ELSD: Liquid chromatography-photodiode-array-evaporative light scattering detector.

**Table 3:** Examples of some selected qualitative, quantitative, and semiquantitative analysis of HMPs.

Types of HMPs	Medicinal value	Type of analysis	Method of analysis	Reference
*Brucea javanica*	Treatment of cancer, amebic dysentery, and malaria	Qualitative analysis	HPLC-QTOF/MS	Tan et al. [93]

*Scutellaria baicalensis* Georgi (Huang-Qin)	Treatment of fevers, ulcers, cancers, and inflammation	Qualitative analysis	HPLC-DAD-MS	Horvath et al. [103]

*Dalbergia odorifera*	Treatment of blood disorders, ischemia, and swelling	Qualitative analysis	HPLC-DAD/ESI-MS/MS	Liu et al. [104]

Sanqi *(Panax notoginseng)*	Hemostatic and cardiovascular properties	Qualitative analysis	HPLC-QTOF/MS	Tan et al. [102]

Radix Angelicae Dahuricae	Anticancer and antibacterial properties	Quantitative analysis	HPLC-ESI-MS/MS	Zheng et al. [105]

*Yucca gloriosa L*	High antioxidant and antiproliferative activities	Quantitative analysis	LC-ESI-MS	Skhirtladze et al. [106]

Shenqi Fuzheng injection (SFI)	Good immunoenhancement and anticancer activity	Quantitative analysis	SPE-HPLC-UV/ELSD	Wang and Qu [107]

Sanqi *(Panax notoginseng)*	Hemostatic and cardiovascular properties	Quantitative analysis	HPLC-QTOF/MS	Tan et al. [102]

Rhubarb *(Rhei rhizome)*	Cathartic and laxative properties	Semiquantitative analysis	HPLC-DAD	Gao et al. [108]

*Fritillaria*	Treatment of chronic cough, lungs cancer	Semiquantitative analysis	LC/ESI-TOF-MS	Zhou et al. [109]

*Brucea javanica*	Treatment of cancer, amebic dysentery, and malaria	Semiquantitative analysis	HPLC-ELSD	Tan et al. [110]

*Triticum durum*	Antioxidant, anti-inflammatory, antimicrobial, and anticancer	Semiquantitative analysis	LC/ESI-MS	Cavaliere et al. [111]

HPLC-DAD: liquid chromatography-diode-array detector; HPLC-DAD-MS: liquid chromatography-diode-array detector-mass spectrometry; HPLC-DAD/ESI-MS/MS: liquid chromatography-diode-array detector/electrospray ionization-mass spectrometry/mass spectrometry; HPLC–ESI-MS/MS: liquid chromatography-electrospray ionization-mass spectrometry/mass spectrometry; HPLC-DAD-TOF/MS: liquid chromatography-diode-array detector-time-of-flight-mass spectrometry; HPLC-DAD-ESI-MS: liquid chromatography-diode-array detector-electrospray ionization-mass spectrometry; HPLC-QTOF/MS: liquid chromatography-quadrupole time-of-flight- mass spectrometry; LC-MS/MS: liquid chromatography-mass spectrometry-mass spectrometry; LC/ESI-TOF-MS: liquid** c**hromatography/electrospray ionization time of flight mass spectrometry; SPE-HPLC-UV/ELSD: solid-phase extraction, high performance liquid chromatography, and ultraviolet/evaporative light scattering detection.

**Table 4:** Example of some chemometric methods used for the analysis of HMPs.

Types of HMPs	Medicinal value	Methods of analysis	Chemometrics method	Reference
Basil (*Ocimum *sp.)	Antimicrobial and antioxidant properties	HPLC-DAD	PCA	Grayer et al. [112]

Radix Isatidis *(Isatis indigotica)*	Influenza, epidemic hepatitis, and epidemic encephalitis B	RP-HPLC	HCA	Zou et al. [73]

*Artemisia selengensis *Turcz.	Diminishing inflammation, relieving a cough, and stimulating the appetite	HPLC–PAD	PCA, HCA	Peng et al. [113]

Flos Lonicerae Japonicae	Treatment of bacterial and virus diseases, inflammation, and fever	HPLC-DAD	HCA	Li et al. [114]

*Polygala japonica *Houtt.	Anti-inflammatory, antibacterial, and antidepressant agent	HPLC-DAD-ELSD	PCA	Hong-Lan et al. [115]

*Cassia obtusifolia* L. or *Cassia tora*	Improving eyesight and medicinal values as a cathartic and diuretic	HPLC-DAD	PCA, PLS, BP-ANN, and RBF-ANN	Lai et al. [116]

*Ocimum americanum, O. citriodorum*	Strong fungicidal activity, antioxidant activity	HPLC-DAD	PCA	Vieira et al. [117]

*Ziziphus jujuba *Mill	Immunity stimulant and antitumour activities	HPLC-DAD	PCA, HCA	Guo et al. [118]

*Fructus Aurantii Immaturus*	Eliminating sputum and dispersing painful abdominal mass	HPLC-DAD	PCA, HCA, SA	Xu et al. [87]

PCA: principal component analysis; HCA: hierarchical clustering analysis; PLS: partial least squares; BP-ANN: back propagation artificial neural network; RBF-ANN: radial basis function artificial neural network; HPLC-DAD: liquid chromatography-diode-array detector; HPLC-PDA: liquid chromatography photodiode-array; HPLC-DAD-ELSD: liquid chromatography-diode-array detector-evaporative light scattering detection; HPLC-APCI-MS: liquid chromatography–atmospheric-pressure chemical ionization-mass spectrometry; RP-HPLC: reverse-phase liquid chromatography; LDA: linear discriminant analysis; SA: similarity analysis; PLS: partial least square.

**Table 5:** Different DNA extraction methods that have been used in HMPs.

Method used	Extraction method	Reference/supplier	Medicinal parts/type of HMPs	References
			Roots, leaves, powder, stem, seeds, whole plant	Vassou et al. [47]
			Leaves and fruit	Srirama et al. [119]
			Juice	Mahadani and Ghosh [120]
			Leaves	da Costa et al. [121]
			Fruits, stem, seeds, herb, cortex, flowers, rhizome	Han et al. [27]
Modified CTAB methods	Salting out precipitation	Doyle [122]	Herb	Kumar et al. [123]
			Leaves	Alexander [124]
			Herb	Li et al. [125]
			Leaves	Costa et al. [126]
			Dry leaves, bark, fruit	Seethapathy et al. [127]
			Leaves	Selvaraj et al. [128]
			Powder	Sheth and Thaker [129]
			Herbs	Cheng et al. [130]
			Roots	Zhou et al. [131]
			Rhizome	Wong et al. [132]

Modified SDS methods	Salting out precipitation		Leaves, flower, bud	Tamari et al. [133]
		Edwards et al. [134]	Leaves	Alexander [124]
			Leaves	da Costa et al. [121]
			Herbs	Cheng et al. (2014)

Wizard Genomic DNA purification kit	Silica binding	Promega	Tea	Jian et al. [135]

			Capsule, tablets, powder, leaf	Newmaster et al. [20]
			Dried and fresh plant tissue, capsule, tea, dried bark	Singtonat and Osathanunkul [136]
NucleoSpin plant II mini	Silica binding	Macherey-Nagel	Tea	Uncu et al. [137]
			Plant tissue	Osathanunkul et al. [138]
			Seeds	Costa et al. [126]
			Dried plant tissue	Osathanunkul et al. [139]
			Leaves, pills, cone, stem, roots, bark	Llongueras et al. [140]

NuceloSpin Tissue Kits	Silica binding	Macherey-Nagel	Tea, roots, capsule, liquid, root pieces, tablets	Wallace et al. [46]

			Bark	Palhares et al. [141]
			Capsule, tablets	Baker [142]
			Leaves	da Costa et al. [121]
			Leaves, flower, roots	Palhares et al. [143]
DNeasy Plant mini kit	Silica binding	Qiagen	Leaves, pills, cone, stem, roots, bark	Llongueras et al. [140]
			Powder	Cimino [144]
			Leaves	Alexander [124]
			Leaves	Enan and Ahmed [145]
			Fruit, powder	Parvathy et al. [146]

DNeasy96 Plant kit	Silica binding	Qiagen	Tea	Stoeckle et al. [147]

Plant Genomic DNA Kit	Silica binding	Tiangen Biotech	Decoction	Xin et al. [148]
			Dried bulbus	Xiang et al. [149]
			Leaves, cortex	Zhang et al. [150]
			Crude drug, dry leaf, root	Zhu et al. [151]
			Root, pills, capsule, tablets	Liu et al. [152]
			Dry fruit, leaves	Dian-Yun et al. [153]
			Dry leaves, roots	Hu et al. [154]
			Dry flower bud	Hou et al. [155]

IBI genomic DNA mini kit (plant, GP1)	Silica binding	IBI	Leaves, pills, cone, stem, roots, bark	Llongueras et al. [140]

**Table 6:** Overview of chemical and DNA barcoding methods for herbal medicinal plant identification.

Analysed material	Chemical methods applied	Genomic region analysed	References
*H. virginiana, M. recutita, M. ilicifolia, M. glomerata, P. ginseng C., P. incarnate, P. boldus, and V. officinalis*	HPLC	*rbc*L, *mat*K, and ITS2	Palhares et al. [143]

*Solanum lyratum* and *Aristolochia mollissima*	HPLC and TLC	*rbc*L, *mat*K, ITS2*, trn*H*-psb*A, and *trn*L*-trn*F	Li et al. [125]

*Hedyotis diffusa*	HPLC and TLC	ITS2	Li et al. [156]

*Cinchona pubescens*	HPLC and TLC	*rbc*L and *mat*K	Palhares et al. [141]

*Salvia L.*	HPLC	ITS2	Jian-ping et al. [157]
